# Effect of Sintering Conditions on the Mechanical Strength of Cu-Sintered Joints for High-Power Applications

**DOI:** 10.3390/ma11112105

**Published:** 2018-10-26

**Authors:** Jeong-Won Yoon, Jong-Hoon Back

**Affiliations:** 1 Welding and Joining R&D Group, Korea Institute of Industrial Technology (KITECH), 156 Gaetbeol-ro, Yeonsu-gu, Incheon 21999, Korea; bjh1218@kitech.re.kr; 2 Critical Materials and Semiconductor Packaging Engineering, University of Science and Technology (UST), 217 Gajeong-ro, Yuseong-gu, Daejeon 34113, Korea

**Keywords:** Cu paste sintering, Cu nano powder, power electronics, sinter-bonding, shear strength

## Abstract

In this study, the feasibility of low-cost Cu-sintering technology for power electronics packaging and the effect of sintering conditions on the bonding strength of the Cu-sintered joint have been evaluated. A Cu paste with nano-sized Cu powders and a metal content of ~78% as a high-temperature bonding material was fabricated. The sinter-bonding reactions and mechanical strengths of Cu-sintered joints were evaluated at different sinter bonding pressures, temperatures, and durations during the sintering process. The shear strength of the Cu-sintered joints increased with increasing sintering pressure. Good interfacial uniformity and stable metallurgical microstructures were observed in the Cu joints sintered at a high sintering pressure of 10 MPa, irrespective of the sintering time. It was confirmed that a high-pressure-assisted sintering process could create relatively dense sintered layers and good interfacial uniformity in the Cu-sintered joints, regardless of the sintering temperatures being in the range of 225–300 °C. The influence of the sinter bonding pressure on the shear strengths of the Cu-sintered joints was more significant compared to that of the sintering temperature. Durations of 10 min (at 300 °C) and 60 min (at 225 and 250 °C) are sufficient for complete sintering reactions between the Si chip and the direct bond copper (DBC) substrate. Relatively good metallic bonding and dense sintered microstructures created by a high sintering pressure of 10 MPa resulted in high shear strength in excess of 40 MPa of the Cu-sintered joints.

## 1. Introduction

Of late, there has been considerable interest in improving technology for electric vehicles (EV) and hybrid EVs (HEV) due to global environmental issues. A main component of any power electronic system in EV/HEV is the inverter power module, which contains power semiconductor devices, die-attached materials, ceramic substrate or lead-frame, and bonding/interconnection materials [1,2]. Among them, die-attachment or chip bonding plays an important role in current power electronic systems because the chip bonding material directly interfaces with the semiconductor chips and ceramic substrates. The maximum temperature of the chip is extremely high during operation of the power electronic systems, e.g., modules in EV/HEV. Especially, the operation temperatures exceeded 200 °C, when silicon carbide (SiC) and gallium nitride (GaN) devices are used as a substitutional power chip to the conventional Si device [3,4,5,6,7]. Consequently, the high-temperature endurable reliability levels for such power modules are required, and new die-attach bonding materials and technologies should be used to meet the demands of power modules. Hence, many researchers and manufacturers in the power module industry try to raise the chip operation temperature up to 200 or 300 °C for next-generation power modules. Until now, the conventional soldering method had been the general die-attaching technique for power packaging [8,9,10,11,12,13,14]. Many people have used high-Pb or Pb-free solders for the soldering. However, general solder alloys have relatively low melting points and electrical/thermal conductivities when compared to conductive metals such as Cu and Ag. Especially, they are difficult to use for the long term because of their low melting point, low creep resistance, and the presence of brittle intermetallic compounds at the joining interface [13,14]. One approach is transient liquid phase (TLP) bonding, which was developed by combining of conventional soldering and diffusion bonding process [15,16,17,18,19]. The advantages of the TLP bonding process are low cost, high melting points, and moderate mechanical strength. On the other hand, the drawbacks of this process include the brittle nature of intermetallic compounds and their unidentified reliability. One of the die-attaching methods that can replace conventional soldering is Ag sintering technology [20,21,22,23,24,25,26,27]. Ag sintering technology is being considered to replace high-Pb solders for application in high temperature power electronics, because of stability at high temperatures, good thermal and electrical conductivity, and long-term reliability. Nano or micro-sized Ag powders are mixed with organic materials such as thinners, binders, and dispersants to form Ag pastes. However, high cost is a drawback of this process due to the use of a precious Ag metal. To overcome the drawback of the Ag sintering, many researchers have been evaluated Cu-sintering technology [28,29,30,31,32,33,34]. Cu has many advantages, such as low cost, low electrical resistivity, high thermal conductivity, and stronger durability towards ion migration when compared to Ag [28]. Nevertheless, there is insufficient data and reports on the study of sintering conditions and joint strength between the Cu-sintered layer and chip/substrate. We fabricated a micro-sized Ag paste and studied the sintering bonding reactions and joint strengths of the Ag sintered joints with various surface finishes [35]. In the study, we confirmed that enough sinter bonding reactions and relatively stable microstructures were formed on the Cu substrate, even though the Ag sintered joint on the Cu substrate had a lowest shear strength value of about 30 MPa, compared to Ag and electroless Ni-immersion Au (ENIG) finished substrate. Therefore, in this study, the feasibility of low-cost Cu-sintering as a die-attaching method for power electronics packaging has been evaluated with Ag-finished chip and substrate. First, a Cu paste with nano-sized Cu powders was fabricated and used to sinter Si chips for direct bond copper (DBC) substrates. Next, the Cu-sintering process was performed with the fabricated Cu paste to bond between Ag-finished Si chip and DBC substrate. In the sintering process, bonding temperature, time, pressure, and atmospheric conditions are very critical towards determining the sinter-bonding quality in the sintered joints. Mechanical shear tests were conducted to determine the effect of sintering conditions on the shear strength of Cu-sintered joints, and the connections between the microstructures and the joint shear strengths of the Cu-sintered joints have been discussed.

## 2. Materials and Methods

**Figure 1:**
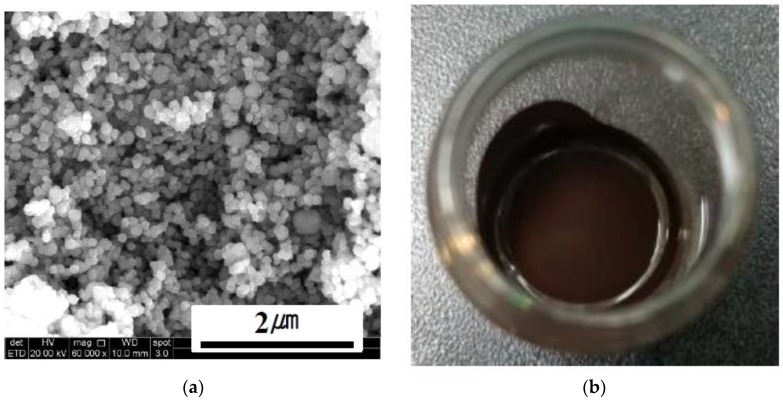
SEM images of the (**a**) Cu nano powders, and (**b**) fabricated Cu paste.

**Figure 2:**
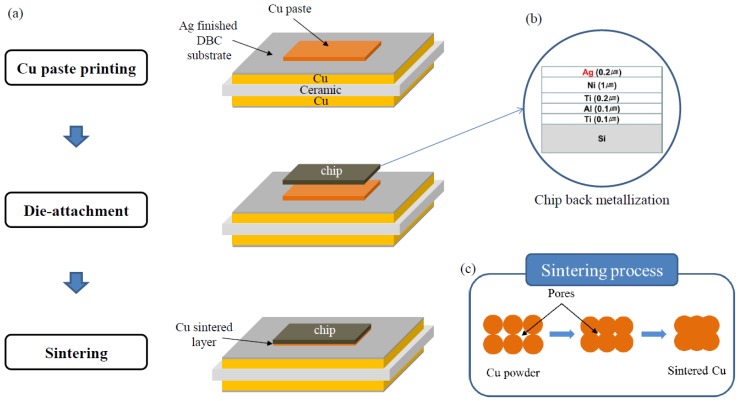
(**a**) Sample fabrication steps used in this study, (**b**) schematic of the chip back metallization and (**c**) schematic of the sintering process.

A Cu paste with nano-sized Cu powders was fabricated as the sinter bonding material. The Cu metal content and particle size were ~78 wt % and ~40 nm, respectively. In order to evaluate oxidation degree of Cu nano powders, the Cu powders were analyzed by X-ray diffraction (XRD, Malvern Panalytical X’PERT PRO, Cu Kα). Poly ethylene glycol (88440-250ML-F, Sigma-Aldrich, St. Louis, MO, USA) was used as a solvent. The Cu powder (CNVISION Co., Ltd., Seoul, Korea) was dispersed in the solvent, and sequentially mixed for 20 min in order to obtain a homogeneous Cu paste. Figure 1 shows the Cu nano powders and the fabricated Cu paste in this study. To investigate thermal characteristic of the fabricated Cu paste, the thermogravimetric analysis (TGA) was performed in a machine (Q50, TA Instruments, New Castle, PA, USA) with a heating rate of 3 °C/min in air. The substrates were direct bond copper (DBC) ceramic substrates, and the dimensions of the DBC substrates were 10 × 10 × 0.98 mm. The DBC substrates are consisted of Cu/96% Al_2_O_3_/Cu sandwiched structure, and the thicknesses of the Al_2_O_3_ ceramic plate and Cu layers were 380 and 300 μm, respectively. And, the DBC substrate was finally coated with Ag layer of approximately 0.3 µm in thickness. The fabricated Cu paste was screen-printed using a 50 µm-thick SS 304 stainless steel mask with an opening of 3 × 3 mm. Subsequently, Si dummy chips (3 mm × 3 mm) metallized with 0.1 µm Ti/0.1 µm Al/1 µm Ni/0.2 µm Ag were placed on the printed Cu paste. Finally, the Cu-sintering process was performed in a die-bonding machine (T-3000-FC3-HF, Tresky, Thalwil, Switzerland) in air atmosphere. Pre-drying was performed for 2 min at 200 °C. The sintering temperatures (225, 250, and 300 °C), durations (10, 30, and 60 min), and pressures (0.5, 2, 5.5, and 10 MPa) were used as bonding parameters in this study. Schematics of the sinter-bonding process, chip back metallization, and sintering mechanism are depicted in Figure 2. After sintering process, the Cu-sintered samples were cooled to room temperature. To evaluate defects such as voids and delamination, X-ray non-destructive testing was performed on the sintered samples using an X-ray inspection machine (XSCAN-H160-OCT, XAVIS, Seongnam, Korea). General metallographic procedures were used to examine the cross-sections of the sintered joints. The interfacial microstructures and chemical compositions were investigated using a scanning electron microscope (SEM, INSPECT F, FEI, Hillsboro, OR, USA) equipped with an energy-dispersive X-ray spectroscope (EDX). In addition, a focused ion beam (FIB, NOVA-600, FEI, Hillsboro, OR, USA) milling machine was used to examine the interfaces of the Si chip/Cu-sintered layer/DBC joint in more detail. A back scattered electron (BSE) image mode of SEM was used to observe more distinguishable boundary lines between different metal layers. In addition, Pt was coated on the samples for SEM observations. To evaluate the bonding strength between chip and DBC substrate, die shear tests were conducted in air atmosphere using a bond tester (DAGE-4000, Nordson DAGE, Aylesbury, UK) with a shear height and speed of 200 μm and 200 μm/s, respectively. Five Cu-sintered joints were tested under each condition and the average shear strength values are reported.

## 3. Results

**Figure 3:**
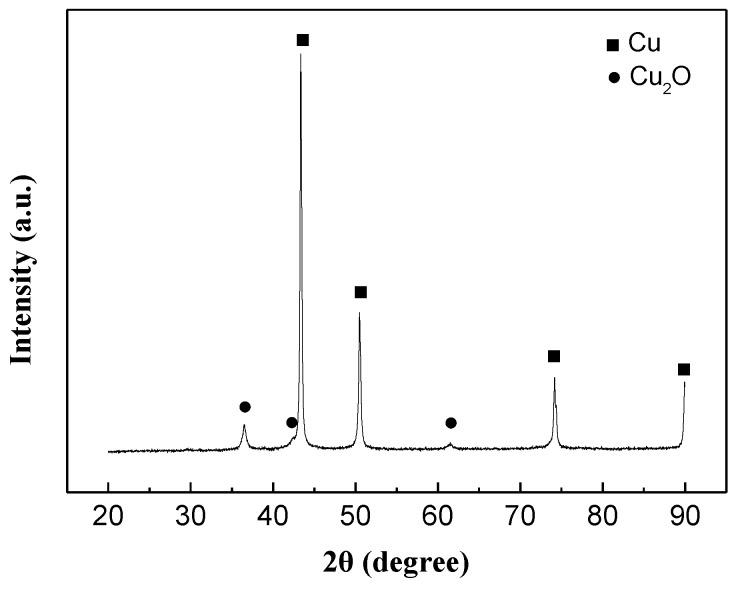
XRD pattern of Cu powders.

In order to evaluate oxidation degree of Cu nano powders, the Cu powders were analyzed by XRD. According to the XRD result (Figure 3), there were four strong peaks (■) at 2θ = 43.3°, 50.5°, 74.1°, and 90°, which are corresponding to Cu. In addition, there were three other relatively weaker peaks (●) at 2θ = 36.5°, 42.6° and 61.5°, which are corresponding to Cu_2_O. Although some relatively weak intensity Cu_2_O peaks were observed, we tried to fabricate Cu paste, because the nano-sized Cu powders are easily oxidized in air.

**Figure 4:**
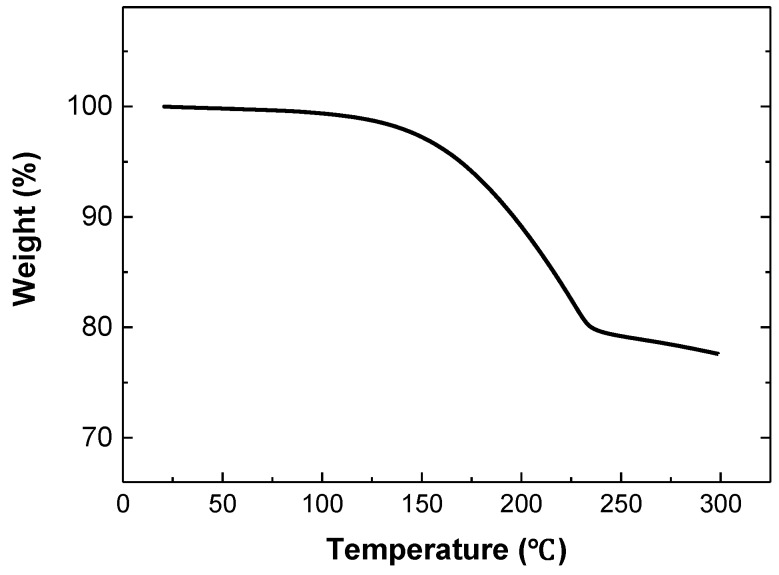
TGA result of the Cu paste.

A Cu paste with nano-sized Cu powders was successfully fabricated. To investigate the thermal property of the fabricated Cu paste, we performed the TGA analysis. Figure 4 shows the TGA analysis result. From the TGA result in the temperature range of 25~300 °C, we confirmed that the weight loss of the Cu paste started at around 150 °C and completed at around 225 °C. These weight losses were related to the evaporation of solvent during heating. After heating up to 300 °C, approximately 78 wt % Cu remained. This content was well matched to the Cu metal content used in the Cu paste fabrication.

**Figure 5:**
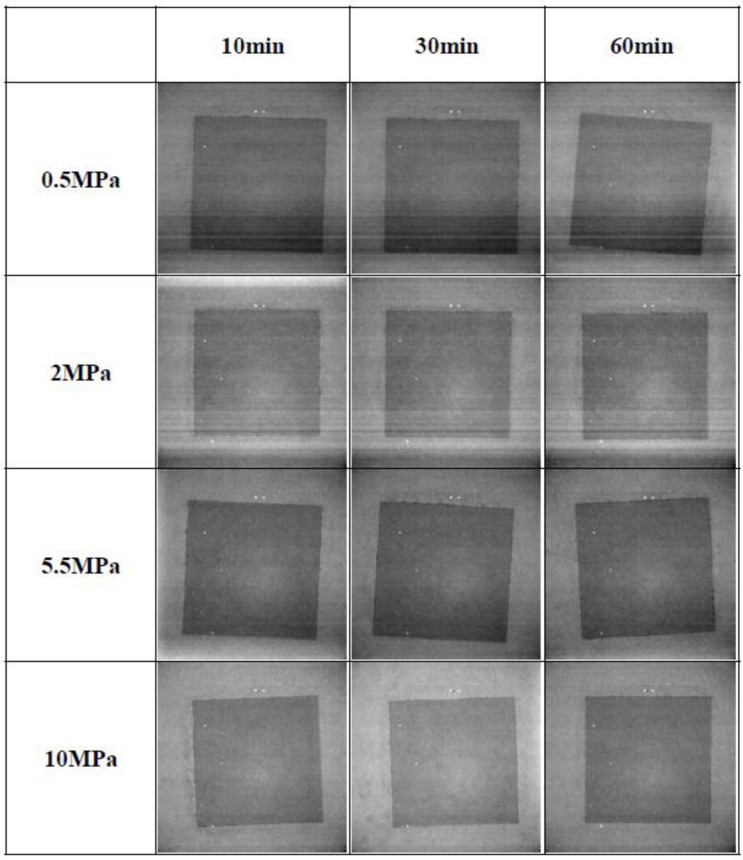
X-ray images of the Cu-sintered joints under various sintering conditions.

The Cu-sintering process was conducted under various sintering conditions. X-ray inspections were performed to observe areas with no bonds and defects such as voids at the Cu-sintered interfaces. Figure 5 shows the X-ray observation images of the Cu-sintered joints at 300 °C under various sintering conditions. There were no big differences between black and white contrasts in the X-ray images. In other words, the uniform levels of the X-ray images suggest that the sintered Cu joints do not have relatively large voids. From the results of the X-ray inspection, it was confirmed that the Cu-sintered joints were fabricated and bonded well, and that there were no distinguishable defects at the interfaces, regardless of the different sintering conditions.

**Figure 6:**
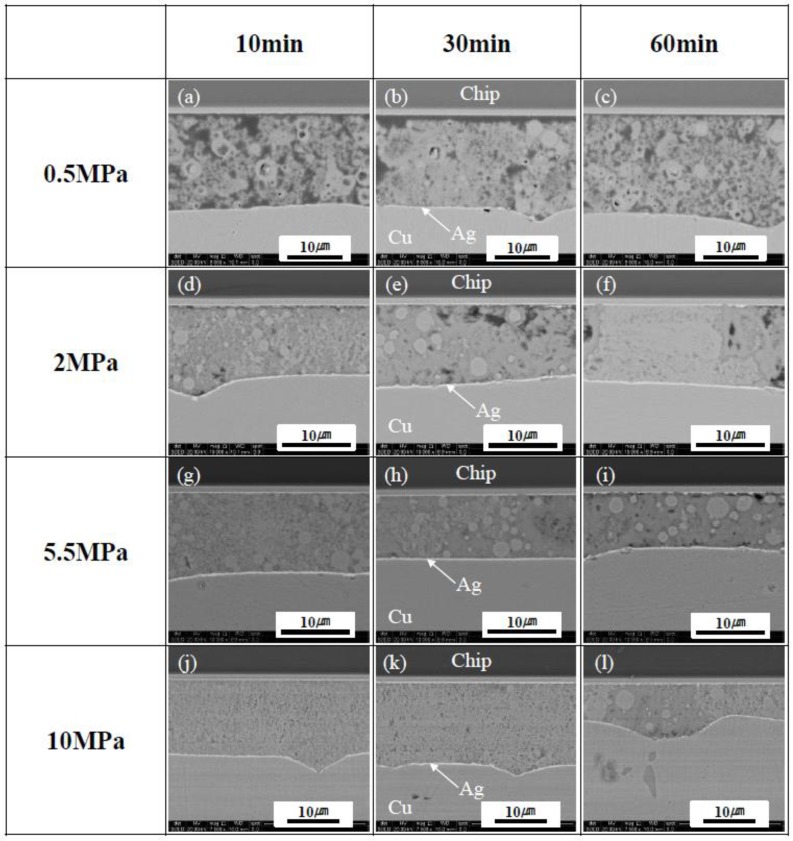
Cross-sectional SEM images of the Si chip/sintered Cu layer/DBC joints at different sintering pressures and durations at 300 °C; (**a**–**c**) 0.5, (**d**–**f**) 2, (**g**–**i**) 5.5, and (**j**–**l**) 10 MPa.

Figure 6 shows the cross-sectional SEM images of the Ag-finished Si chip/sintered Cu layer/DBC joints at different sintering pressures and times at 300 °C. Although large defects or voids were not observed during the inspection of the X-ray, the sinter joints bonded at a low bonding pressure of 0.5 MPa had relatively loose Cu-sintered layers, and sufficient sinter bonding reactions between Cu particles and/or chip/DBC metal layers had not occurred during the sintering process, as shown in Figure 6a–c. In the case of a relatively low bonding pressure of 0.5 MPa, enough contacts between Cu particles and chip or DBC metal layers did not occur, which resulted in the formation of relatively loose Cu-sintered joints. As the sintering pressure was increased to 2 MPa (Figure 6d–f), the sinter bonding effectively occurred at both interfaces, and the Cu-sintered layer satisfactory adhered to both the Ag finish layers of the Si chip and the DBC substrate. As the sintering pressure increased up to 5.5 and 10 MPa, more stable interfacial microstructures and relatively dense sintered layers were observed in the Cu-sintered joints, as shown in Figure 6g–l. The presence of good interfacial uniformity and metallurgically stable interfacial microstructures observed in the Cu joints sintered at a high bonding pressure of 10 MPa (regardless of the sintering duration) was confirmed. Despite a short sinter-bonding duration of 10 min, a good Cu-sintered joint can be obtained, as shown in Figure 6j. The applied sintering pressure increased the events of contacts between Cu nanoparticles, resulting in increase of sinter-bonding rate and decrease of voids and loose microstructures.

**Figure 7:**
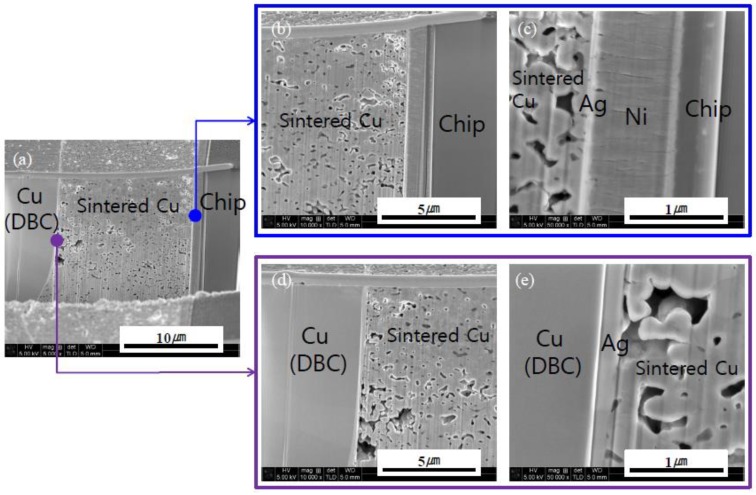
Cross-sectional FIB images of the Si chip/sintered Cu layer/DBC joint (sintering conditions: 300 °C, 10 MPa, 10 min).

FIB analyses were performed to observe the interfacial microstructures of the Cu-sintered sample shown in Figure 6j, whose results can be seen in Figure 7. The Cu-sintered layer between the chip and DBC substrate was clearly observed, and good metallurgical bonding between the Cu particles and Ag layers of both chip and DBC substrates was confirmed at both interfaces. We confirmed the presence of relatively white thin Ag coating layers between sintered Cu and chip or DBC substrate. According to previous literatures [20,25], good metallic bonding occurs at the Ag/Cu interface during sintering. Cu and Ag have the same face-centered-cubic (FCC) crystal structure, and their binary system forms complete solid solutions. They also do not form any intermetallic compounds, and atomic inter-diffusion occurs easily without any significant lattice distortion. Consequently, inter-diffusion occurs between the Cu particles and Ag surface finishes of both chip and DBC substrates during the sintering process, which resulted in relatively good Cu-sintered joints under a bonding pressure of 10 MPa. Some micro-sized sintering voids were also observed in the Cu-sintered joint.

**Figure 8:**
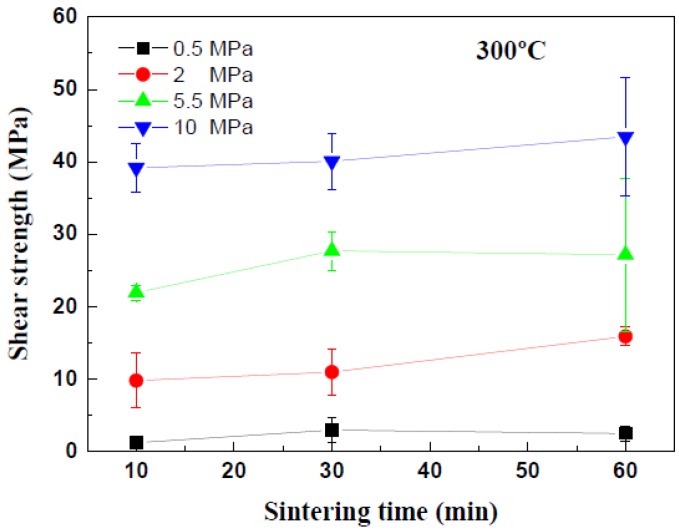
Variation of die shear strength with the sintering pressure and duration.

Die shear tests were performed to evaluate the effects of the sinter bonding conditions on the mechanical strengths of the Cu-sintered joints. Figure 8 shows the variations in the die shear strengths of the Cu-sintered joints with different values of sintering pressure and duration at a temperature of 300 °C. As anticipated from the observations of the interfacial microstructure in Figure 6, the sinter bonding pressure strongly affected the shear strengths of the Cu-sintered joints, i.e., the shear strength of the Cu-sintered joints increased with an increase in sintering pressure. In the case of sintering duration of 10 min, the die shear strengths of the Cu-sintered joints at sintering pressures of 0.5, 2, 5.5, and 10 MPa were approximately 2, 10, 20, and 40 MPa, respectively. As anticipated from Figure 6, low shear strength values were obtained at a low sintering pressure of 0.5 MPa. The highest shear strength which exceeded 40 MPa was obtained at a sintering pressure of 10 MPa. Interestingly, the shear strengths do not significantly increase with an increase in sintering duration, regardless of sintering pressure. In other words, duration time of 10 and 60 min produced Cu-sintered joints with similar shear strength. In this study, a sintering duration of 10 min at 300 °C is sufficient for complete sintering reactions between the chip and the DBC substrate. In other words, the fabricated Cu paste requires a relatively short process time for sinter bonding, which will be beneficial for real-time fabrication in a mass production. These shear strength results are consistent with those observed in the microstructures of the Cu-sinter joints shown in Figure 6. Good metallic bonding and denser sintered microstructures generated by a high sintering pressure resulted in high shear strength of the Cu-sintered joints.

**Figure 9:**
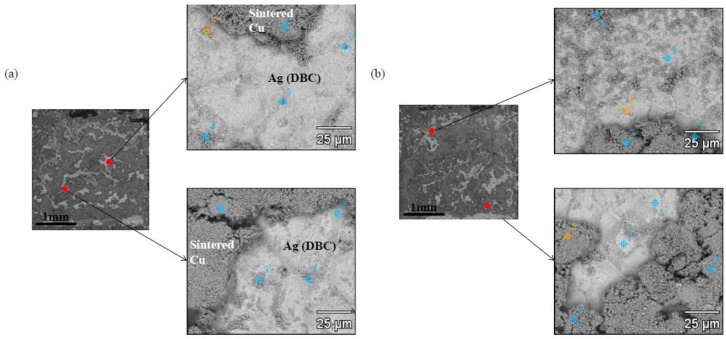
Fracture surfaces of the Cu-sintered joints after die shear testing at: (**a**) 5.5 MPa, 10 min and (**b**) 5.5 MPa, 30 min.

**Figure 10:**
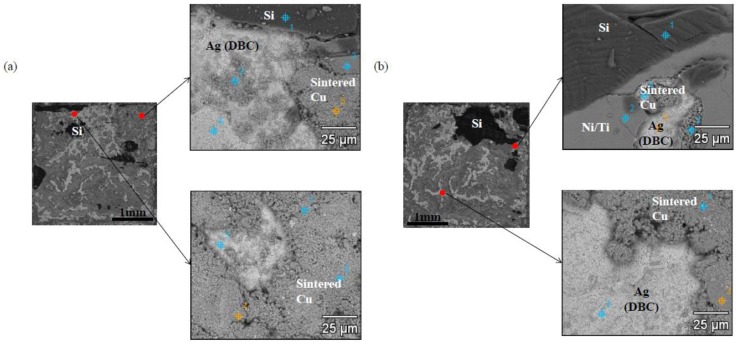
Fracture surfaces of the Cu-sintered joints after die shear testing at: (**a**) 10 MPa, 30 min and (**b**) 10 MPa, 60 min.

To search the reason for the difference in the die shear strengths, the fracture surfaces were investigated and analyzed after shear testing. Figure 9 and Figure 10 show the SEM images of the fracture surfaces of the Cu-sintered joints that bonded at pressures of 5.5 and 10 MPa, respectively. Similar fracture surfaces were observed in the Cu-sintered joints under the bonding pressure regardless of different sintering times. These results of the fractured surfaces were well matched with the shear strength results shown in Figure 8. However, the fracture surfaces in the Cu-sintered samples with different sintering pressure were slightly different. At a sintering pressure of 5.5 MPa, fractures mainly occurred on the sintered Cu layer of the upper side of the Si chip. It was also confirmed that the fracture partially occurred on the Ag-finished DBC substrate. In Figure 9, relatively dark and bright areas denote the sintered Cu layer and Ag-finished DBC, respectively. Relatively dark sintered Cu layers occupied a large portion of the fractured surfaces. From these results, we confirmed that the fractures mainly occurred at the upper interface between the sintered Cu layer and Si chip. On the other hand, Si chips were observed in the fracture surfaces (Figure 10) at a sintering pressure of 10 MPa, indicating higher interfacial adhesion and shear strength. In Figure 10, black areas denote the Si chip. In this case, fractures also occurred primarily at the upper side of the Si chip/Cu-sintered interfaces. Partially bright-colored Ag-finished DBC surfaces were also observed in the fractured surfaces.

**Figure 11:**
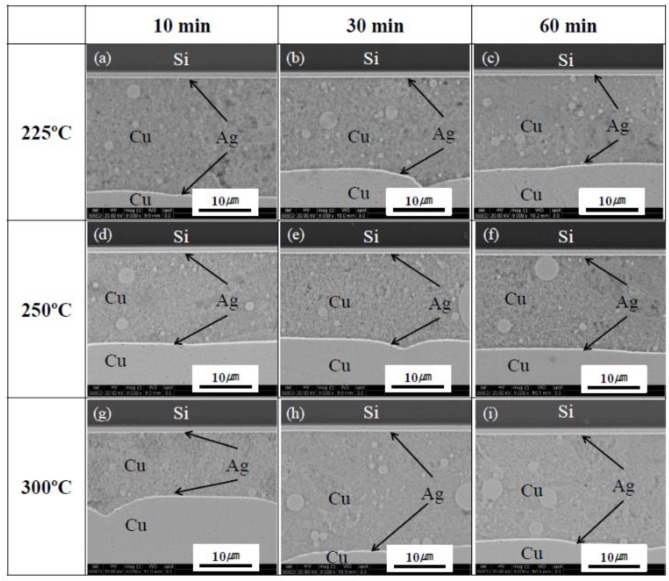
Cross-sectional SEM images of the Si chip/sintered Cu layer/DBC joints at different sintering temperatures and durations (10 MPa); (**a**–**c**) 225, (**d**–**f**) 250, and (**g**–**i**) 300 °C.

To evaluate the effect of sintering temperature on the microstructure and mechanical strength of the Cu-sintered joints, the sintering processes were performed at different temperatures of 225, 250, and 300 °C. Figure 11 shows the cross-sectional SEM images of the Si chip/sintered Cu layer/DBC joints at different sintering temperatures and durations, at a constant pressure of 10 MPa. The high-pressure assisted processes (at 10 MPa) improved the sintering efficiency of Cu particles as a whole. Large defects or voids were not observed in the cross-sectional SEM images, regardless of different sintering temperatures and durations. Unlike low bonding pressures of 0.5 and 2 MPa (shown in Figure 6a–f), sufficient sinter bonding reactions occurred between Cu particles and/or chip/DBC metal layers during the sintering process in spite of low bonding temperatures of 225 and 250 °C, as shown in Figure 11a–f. It was confirmed that a high-pressure assisted sintering process could generate relatively dense sintered layers and good interfacial uniformity in the Cu-sintered joints, irrespective of the sintering temperatures which are in the range of 225~300 °C. In spite of a low sintering temperature of 225 °C, good quality Cu-sintered joints can be obtained as shown in Figure 11a–c. Ishizaki et al. fabricated their Cu paste with approximately 140 nm Cu particle and performed the sintering process for 5 min at 300 and 350 °C [34]. Liu et al. studied on the low-pressure Cu-Cu bonding using in-situ surface-modified microscale Cu particles for power device packaging [36]. In their experiment, the Cu-Cu joint specimens were first pre-heated at 130 °C for 5 min in air to evaporate the solvent, after which they were heated to the bonding temperature of 300 °C for 20 min. Although the used sintering temperatures of 225–300 °C are too low for Cu (melting point: ~1083 °C) in this study, the applied bonding pressure effectively assisted to sinter-bonding and influenced on the shear strength of the Cu-sintered joints. When bonding pressure is applied during sintering process, the hydrostatic pressure and shear stress built up in the Cu-sintered joint [21]. The applied pressure increased the events of contacts between Cu nanoparticles, resulting in increase of sinter-bonding rate. As a result, the path for diffusion between Cu particles and the driving force for Cu-sintering reaction increased. Therefore, we could easily Cu-sinter-bonding under an applied pressure of up to 10 MPa even relatively low sintering temperatures of 225–300 °C in this study.

**Figure 12:**
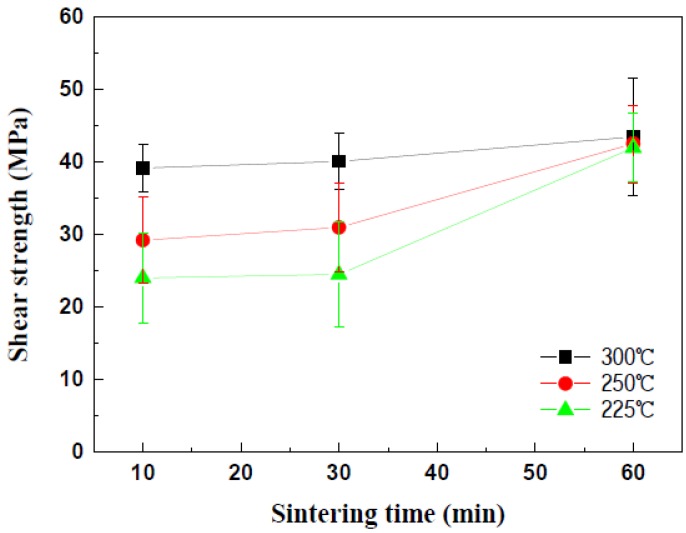
Variation in die shear strength with the sintering temperature and duration.

Die shear tests were performed to evaluate the effects of the sintering temperatures on the mechanical strengths of the Cu-sintered joints, whose results can be seen in Figure 12. In the case of sintering duration for 10 min, the shear strength of the Cu-sintered joints increased with an increase in sintering temperature. The die shear strengths of the Cu-sintered joints at temperatures of 225, 250, and 300 °C were approximately 25, 30, and 40 MPa, respectively. These values do not significantly increase with an increase in sintering duration for up to 30 min, regardless of sintering temperature. Even after the sintering time expanded from 10 min to 30 min, the overall shear strengths remain constant regardless of the sintering temperature. As the sintering time increases to 60 min, the shear strength of the Cu joints sintered at 225 °C and 250 °C increases to approximately 40 MPa. Interestingly, these shear strength values are similar to that at 300 °C. This implies that the sintering reactions at different temperatures have been completed and are almost saturated under these sintering conditions. In this study, the sintering durations of 10 min at 300 °C and 60 min at 225 °C and 250 °C are sufficient for complete sintering reactions between the chip and the DBC substrate. A high shear strength of approximately 40 MPa was obtained for these sintering conditions. Relatively good metallic bonding and dense sintered microstructures from a high sintering pressure of 10 MPa resulted in the high shear strength of the Cu-sintered joints. By comparing Figure 8 and Figure 12, it was concluded that the sinter bonding pressure affected the shear strengths of the Cu-sintered joints more significantly when compared to the sintering temperature.

## 4. Conclusions

The sinter-bonding reactions and mechanical strength of Cu-sintered joints with Ag-surface finished Si chip and DBC substrates were evaluated during the sintering process. The behaviors of the sintered joints under different sintering conditions (temperature, time, and pressure) were compared and the connection between the sintering behavior and the mechanical shear strength was identified. A Cu paste with nano-sized Cu powders was fabricated and the sintering process was performed by using Ag-surface finished Si chip and DBC. From the observations made via cross-sectional SEM and FIB, it was confirmed that relatively stable sintered microstructures and dense Cu-sintered layers were formed in high bonding pressure conditions. Sintering reaction behaviors result in good metallic bonding during the Cu-sintering process. The shear strength increased with an increase in sintering pressure and temperature. In the case of 10 MPa bonding pressure, a short bonding duration of 10 min was sufficient for complete sintering reactions. A sufficient sinter bonding reaction occurred between the Cu particles and/or chip/DBC metal layers during the sintering process in spite of low bonding temperatures of 225 °C and 250 °C. The sinter bonding pressure affected the shear strengths of the Cu-sintered joints more significantly when compared to the sintering temperature.
